# Estimation of expectedness: Predictive accuracy of standard therapy outcomes in randomized phase 3 studies in epithelial ovarian cancer

**DOI:** 10.1002/cncr.29030

**Published:** 2014-10-02

**Authors:** Vincent Castonguay, Michelle K. Wilson, Ivan Diaz‐Padilla, Lisa Wang, Amit M. Oza

**Affiliations:** ^1^ CHU‐de‐QuébecQuebec CityQuebecCanada; ^2^ Division of Medical Oncology and Haematology, Bras Family Drug Development ProgramPrincess Margaret Cancer CentreTorontoOntarioCanada

**Keywords:** randomized, phase 3 trials, epithelial ovarian cancer, endpoints, survival, statistical design

## Abstract

**BACKGROUND:**

The anticipated clinical outcome of the standard/control arm is an important parameter in the design of randomized phase 3 (RP3) trials to properly calculate sample size, power, and study duration. Changing patterns of care or variation in the study population enrolled may lead to a deviation from the initially anticipated outcome. The authors hypothesized that recent changes in patterns of care in epithelial ovarian cancer (EOC) have led to challenges in correctly estimating the outcome of control groups.

**METHODS:**

A systematic review of the literature was conducted for RP3 trials of EOC published between January 2000 and December 2010. The expected outcome of the control arm as well as the actual outcome achieved by this cohort was collected and a ratio (actual‐over‐expected ratio) was calculated. The estimation of outcome was deemed accurate if the outcome of the control arm was between 0.75 to 1.25 times the anticipated outcome.

**RESULTS:**

A total of 35 trials were eligible for analysis. Fifteen trials had survival as the primary endpoint whereas 20 had a progression‐based primary endpoint. In total, 12 of 15 trials with a survival‐based endpoint significantly underestimated the outcome of the control arm, whereas only 4 of 20 trials with a progression‐based endpoint did. Studies with a survival endpoint underestimated outcome more frequently than those with a progression endpoint (*P*<.001).

**CONCLUSIONS:**

Survival of the control arm has frequently been underestimated in recent EOC RP3 trials. This underestimation means that the initial statistical assumptions of these trials may have been inaccurate. Underestimating the outcome of the control arm may result in trials being underpowered to demonstrate the absolute benefit they were designed to show. ***Cancer* 2015;121:413–422.** © *2014 American Cancer Society*.

## Abstract 2

The anticipated clinical outcome of the standard/control arm is an important parameter in the design of randomized phase 3 trials for the accurate calculation of sample size, power, and study duration but is often underestimated in ovarian cancer trials. Changing patterns of care and variations in enrolled study populations may result in a deviation from the anticipated outcome and subsequent inaccurate statistical assumptions.

## INTRODUCTION

When designing a randomized phase 3 (RP3) trial, an appropriate sample size calculation is necessary to ensure adequate study power to demonstrate a statistically significant difference between the experimental and standard arms. The majority of trial designs estimate the required sample size by incorporating 2 variables: the expected outcome for the standard/control arm and the size effect hypothesized for the experimental treatment or, in other words, the magnitude of the benefit the experimental arm is expected to confer relative to the standard arm.^1^ Thus, accurate estimation of the control arm outcome is necessary in order for initial sample size calculations to be precise and reliable.

The basic concepts of trial design evolve from the initial selection of the null hypothesis and the alternative hypothesis. The consequent treatment effect is intimately related and integral to the calculation of the sample size to achieve adequate statistical power.^2^ Type I (α) errors and type II (β) errors are central concepts in this process.^2^ A type I error is the probability of rejecting the null hypothesis when in fact it is true: a false‐positive result.^2^ This in general is set at a low value (conventionally 0.05).^2^ A type II error is the probability of accepting the null hypothesis when in fact it is false: a false‐negative result.^2^ The power of the study reflects the probability of correctly rejecting the null hypothesis (1‐β).^2^ Sample size calculation is an exercise in determining the number of participants required to simultaneously achieve both the desired power and type I error.^3^ Although this is a simplistic explanation of the concepts of trial design and neglects many of the important intricacies, it emphasizes that inaccurate estimation of any of these components potentially compromises the ultimate results of the trial, even before it has started.

The expected outcome in the control arm is generally inferred from completed clinical trials, using historical data from comparable patient populations treated with similar therapies. However, it is recognized that correctly estimating the outcome of a contemporaneous population is difficult, because variations in patient population, changes in treatment patterns, and random error can result in a significant deviation from even the most robust historical data.^4^

Estimating the outcome of women treated for advanced‐stage epithelial ovarian cancer (EOC) may be particularly challenging given the surgical and therapeutic advances made over the last decades, which have significantly influenced patient outcomes. Up until the early 1980s, standard‐of‐care chemotherapy for patients with advanced‐stage EOC was cyclophosphamide and doxorubicin. In <30 years, platinum agents, taxanes, and several other chemotherapeutic drugs have been incorporated into the routine care of patients with EOC. This coupled with improved access to standard therapy and surgery has resulted in survival gains both in the first‐line setting and among patients with recurrent disease. In contrast to trials performed in the early 1980s, in which the median survival in phase 2 and 3 trials ranged from 15 to 24 months,^5^, ^6^ the median survival times published in the last decade have ranged from 36 to 40 months.^7^, ^8^

We hypothesized that this improvement in reported survival in conjunction with changes in treatment patterns and standard of care has led to challenges when estimating the expected control arm outcome in RP3 trials reported within the past decade. To test this hypothesis, a systematic literature review was conducted to assess the accuracy of control arm outcome predictions in RP3 trials of patients with advanced‐stage EOC that were published or reported from 2000 through 2010.

## MATERIALS AND METHODS

### Search Strategy

A search in MEDLINE and EMBASE was conducted for studies published in the English language between January 2000 and December 2010. Medical Subject Headings (MeSH) terms were “random allocation” AND “ovarian neoplasm.” Keywords were (“ovarian” AND “neoplasm”) OR “ovarian neoplasm” OR (“ovarian” and “cancer”) OR “ovarian cancer” AND (“random” AND “allocation”) OR “random allocation” OR “randomized.”

Citation lists of relevant publications were also reviewed for articles that might have been missed with the search strategy. Finally, abstracts from the annual meetings of the American Society of Clinical Oncology (ASCO) and the European Society for Medical Oncology (ESMO) from 2008 through 2010 were reviewed to include reported yet unpublished trials.

### Study Selection

Trials with the following characteristics were included: RP3 clinical trials performed among patients with EOC comparing 1 systemic treatment (experimental arm) over another (control arm). Only trials in which the primary outcome was overall survival (OS), progression‐free survival (PFS), or time to disease progression (TTP) were included. Trials reporting different primary endpoints (eg, response rate, quality of life) were excluded. Trials had to state explicitly the expected outcome of the control arm used for sample size calculation (either in the publication or in an appendix) and the actual or estimated outcome of the control arm once the study was finished. Trials with a non‐inferiority design were excluded.

### Data Analysis

Trials were screened for eligibility and data were collected using standardized collection forms. Data retrieved included publication details, methodological components, and trial characteristics such as sample size, interventions, and outcome measures. The expected control arm outcome used for sample size calculation, hypothesized experimental arm outcome, and finally the actual or estimated result achieved by the control arm in the trial was recorded. To allow analysis, whenever the expected or actual result was stated as a percentage of patients at a given time point, the result was transformed to a median using an exponential model. If the expected outcome of the control arm and sample size were modified at an interim analysis because of significant imprecision, the parameters used at the time of the initial trial design were used for the primary analysis. However, the revised interim parameters were collected for a separate analysis.

### Statistical Analysis

A simple ratio of the actual (A) outcome of the control arm for the primary endpoint divided by the expected (E) outcome used for sample size calculation was calculated (the A/E ratio). When the expected outcome was stated as a median, a ratio of the A/E median was calculated. When the expected outcome was stated as a percentage of patients at a precise time point, this was transformed to a median result assuming an exponential distribution. For example, if the 5‐year survival rate was reported to be 40%, this was transformed to a median using the following calculation: median = (log (0.5)/log(0.4))*5 = 3.8. In this scenario, the median survival would have been reported to be 3.8 years. This allowed for the calculation of a median when only 1 survival rate was known at a certain time point. Once the median value was calculated, it was used to determine the A/E ratio.

An A/E ratio of 0.75 to 1.25 was defined as reflecting an accurate prediction. This was based on the premise that a 25% difference in the actual versus expected outcome was likely to be clinically and statistically relevant. It was believed that this degree of imprecision was sufficient to cause inaccurate initial power calculations. Thus, a ratio of >1.25 was considered as underestimation whereas a ratio of <0.75 was termed an overestimation. For analysis, studies were stratified by whether the primary endpoint was survival‐based (OS) or progression‐based (PFS or TTP).

A Wilcoxon 2‐sample test was used to compare the A/E ratios of survival‐based trials with those of progression‐based trials to assess whether one of the 2 strata tended to be more inaccurate than the other. The significance level was set at an alpha error of ≤.05.

## RESULTS

### Included Studies

**Figure 1:**
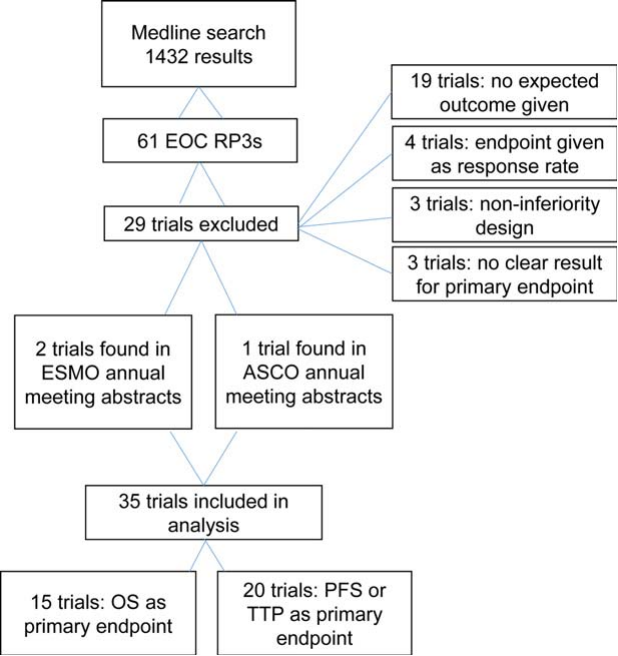
Search results for eligible phase 3 trials of epithelial ovarian cancer (EOC) performed between January 2000 and December 2010 are shown. RP3s indicates randomized phase 3 trials; ESMO, European Society for Medical Oncology; ASCO, American Society of Clinical Oncology; OS, overall survival; PFS, progression‐free survival; TTP, time to disease progression.

With the described search strategy, a total of 61 RP3 trials of systemic therapy in patients with EOC were identified. Twenty‐nine trials were excluded: 19 did not explicitly state the expected control arm outcome in their methods, 4 used response rate as a primary endpoint, 3 trials had a non‐inferiority design, and 3 trials did not clearly report the primary endpoint result.^9^, ^10^, ^11^ A total of 32 trials met all prerequired criteria and were included in the current analysis. In addition, 3 additional unpublished trials met all eligibility criteria: 2 from the ESMO annual meeting abstracts and 1 from the ASCO annual meeting abstracts.^12^, ^13^, ^14^ A total of 35 trials met all prespecified criteria and were included in the analysis (Fig. 1). Of these 35 trials, 15 had OS as a primary endpoint and 20 had either PFS or TTP as a primary endpoint.

For 10 trials, the outcomes for the expected and/or actual primary endpoint were transformed from a percentage of patients alive at a fixed time to a median survival assuming an exponential distribution.^15^, ^16^, ^17^, ^18^, ^19^, ^20^, ^21^, ^22^, ^23^, ^24^ This was then used to calculate the A/E ratio.

### Trials With a Survival‐Based Primary Endpoint

**Figure 2:**
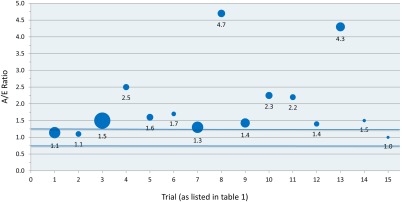
Actual‐over‐expected ratios (A/E) for trials with overall survival as a primary endpoint are shown. The size of the bubble is proportional to the sample size of the control cohort. Blue lines delineate the region between 0.75 and 1.25, termed as being an accurate estimation.

Fifteen trials with OS as a primary endpoint met all criteria (Table 1).^13^, ^15^, ^16^, ^18^, ^19^, ^20^, ^21^, ^22^, ^23^, ^24^, ^25^, ^26^, ^27^, ^28^, ^29^ Twelve trials underestimated the actual OS as defined by an A/E ratio >1.25, whereas 3 trials were accurate in predicting OS as defined by an A/E ratio of 0.75 to 1.25. None of the trials overestimated the actual OS. The range of A/E calculated was from 1.0 to 4.7, with no trial having an A/E ratio of <1 (Fig. 2). The mean and median of all ratios was 2.0 and 1.5, respectively.

**Table 1:** Actual‐Over‐Expected Ratio for Trials Using OS as a Primary Endpoint

Trial No.	Reference	Setting	Control Arm	Experimental Arm	Expected OS for Control Arm	Actual OS	Sample Size of Control Arm	Size Effect Studied	A/E Ratio
1	du Bois 2006^25^	First‐line	Paclitaxel and carboplatin	Paclitaxel, carboplatin, and epirubicin	36 mo (median)	41 mo	635	1.2	1.1
2	Spriggs 2007^26^	First‐line	Cisplatin and paclitaxel (24 h)	Cisplatin and paclitaxel (96 h)	27 mo (median)	29.9 mo	140	1.3	1.1
3	ICON Group 2002^20^	First‐line	Carboplatin or cyclophosphamide, doxorubicin, and cisplatin	Paclitaxel and carboplatin	50% (2‐y OS)	63% (2‐y OS)	1364	1.1	1.5
4	Bolis 2010^19^	First‐line	Paclitaxel and carboplatin	Paclitaxel, carboplatin, and topotecan	20% (3‐y OS)	53% (3‐y OS)	172	1.8	2.5
5	Bolis 2004^18^	First‐line	Cisplatin and paclitaxel (175 mg/m^2^)	Cisplatin and paclitaxel (225 mg/m^2^)	30% (4‐y OS)	46% (4‐y OS)	207	1.3	1.6
6	Ray‐Coquard 2007^23^	First‐line	Cyclophosphamide (500 mg/m^2^), epirubicin, and cisplatin	Cyclophosphamide (1800 mg/m^2^), epirubicin, cisplatin, and G‐CSF	50% (2‐y OS)	66% (2‐y OS)	85	1.3	1.7
7	Pfisterer 2006^22^	First‐line maintenance	Observation	Topotecan maintenance	50% (3‐y OS)	58% (3‐y OS)	650	1.2	1.3
8	Rustin 2010^24^	Recurrence	Delayed treatment^a^	Early treatment^a^	5% (2‐y OS)	53% (2‐y OS)	264	3.0	4.7
9	Colombo 2010^13^	Recurrence	Pegylated liposomal doxorubicin	Patupilone	8.9 mo (median)	12.7 mo	416	1.3	1.4
10	Vergote 2009^27^	Recurrence	Pegylated liposomal doxorubicin or topotecan	Canfosfamide	6 mo (median)	13.5 mo	229	1.4	2.3
11	Hall 2004^28^	Recurrence	Observation	Interferon	15 mo (median)	33 mo	151	1.5	2.2
12	Meier 2009^16^	Recurrence	Treosulfan	Topotecan	55% at 6 mo (median, 6.7 mo)	9.5 mo	119	1.3	1.4
13	Parmar 2003^21^	Recurrence	Any platinum‐based therapy	Paclitaxel and carboplatin	5% (2‐y OS)	50% (2‐y OS)	392	1.2	4.3
14	du Bois 2002^15^	Recurrence	Leuprolide	Treosulfan	40% (6‐mo OS) (median, 4.8 mo)	6.9 mo	39	1.5	1.5
15	Alberts 2008^29^	Recurrence	Carboplatin	Carboplatin and pegylated liposomal doxorubicin	18 mo (median)	18 mo	30	1.3	1.0

Abbreviations: A/E, actual‐over‐expected ratio; G‐CSF, granulocyte‐colony‐stimulating factor; ICON, International Collaborative Ovarian Neoplasm Group; OS, overall survival.

Detailed is expected survival of the control arm used for statistical calculations and actual survival achieved by this arm as well as size effect expected from the experimental treatment. Finally, the actual‐over‐expected ratio for survival of the control arm is given. A/E ratios were rounded to one decimal place.

a This trial was designed to determine if there was a survival benefit with early treatment of relapse based on an elevated CA125 concentration alone.

In 2 of these 15 trials, survival was severely underestimated, as reflected by A/E ratios of 4.3 and 4.7, respectively.^21^, ^24^ In both of these trials, the expected survival in the control arm was revised in interim protocol amendments. This resulted in more precise estimations (A/E ratio of 1.1 and 1.0, respectively). When using these revised parameters, the mean and median of all ratios still remained elevated at 1.5 and 1.4, respectively.

Of the 15 trials, 10 underestimated the control arm outcome by such a margin that the control arm did better than hypothesis for the experimental arm. This highlights the degree of the underestimation.

### Trials With a Progression‐Based Primary Endpoint

**Figure 3:**
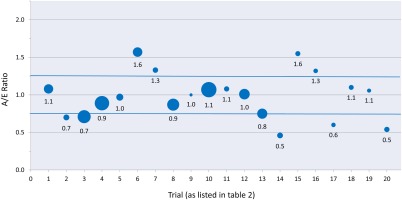
Actual‐over‐expected ratios (A/E) for trials using progression‐free survival and time to disease progression as a primary endpoint are shown. The size of the bubble is proportional to the sample size of the control cohort. Blue lines delineate the region between 0.75 and 1.25, termed as being an accurate estimation of the endpoint.

Twenty trials met all criteria with either PFS or TTP as a primary endpoint. Four trials underestimated the actual outcome (A/E ratio of >1.25), Eleven trials were accurate (A/E ratio of 0.75‐1.25), and 5 trials overestimated the outcome (A/E ratio of 0.75) (Table 2).^12^, ^14^, ^17^, ^30^, ^31^, ^32^, ^33^, ^34^, ^35^, ^36^, ^37^, ^38^, ^39^, ^40^, ^41^, ^42^, ^43^, ^44^, ^45^, ^46^ The A/E ratios ranged from 0.5 to 1.6, with a mean and median ratio of 1.0 and 1.0, respectively (Fig. 3).

**Table 2:** Actual‐Over‐Expected Ratio for Trials Using PFS or TTP as a Primary Endpoint

Trial No.	Reference	Setting	Control Arm	Experimental Arm	Expected PFS in Control Arm)	Actual PFS	Sample Size in Control Arm	Size Effect	A/E Ratio
1	Katsumata 2009^30^	First‐line	Paclitaxel and carboplatin	Dose‐dense paclitaxel and carboplatin	16 mo (median)	17.2 mo	320	1.4	1.1
2	Markman 2009^31^	First‐line maintenance	Paclitaxel management (3 cycles)	Paclitaxel maintenance (12 cycles)	20 mo (median)	14 mo	128	1.3	0.7
3	Burger 2010^12^	First‐line	Paclitaxel and carboplatin	Paclitaxel, carboplatin, and bevacizumab	14 mo (median)	10 mo	625	1.3	0.7
4	Perren 2010^14^	First‐line	Paclitaxel and carboplatin	Paclitaxel, carboplatin, and bevacizumab	18 mo (median)	16 mo	764	1.3	0.9
5	Pfisterer 2006^32^	Recurrence	Carboplatin	Carboplatin and gemcitabine	6 mo (median)	5.8 mo	178	1.4	1.0
6	Monk 2010^33^	Recurrence	Pegylated liposomal doxorubicin	Pegylated liposomal doxorubicin and trabectedin	3.7 mo (median)	5.8 mo	335	1.3	1.6
7	Neijt 2000^34^	First‐line	Paclitaxel and cisplatin	Paclitaxel and carboplatin	12 mo (median)	16 mo	108	1.7	1.3
8	Vasey 2004^35^	First‐line	Paclitaxel and carboplatin	Docetaxel and carboplatin	17 mo (median)	14.8 mo	538	1.25	0.9
9	Papadimitriou 2008^36^	First‐line	No further treatment	High‐dose melphalan	18 mo (median)^a^	18 mo^a^	43	2.0	1.0
10	Bookman 2009^37^	First‐line	Paclitaxel and carboplatin	Paclitaxel, carboplatin and a 3rd agent^b^	15 mo (median)	16 mo	864	1.25	1.1
11	Pecorelli 2009^38^	First‐line maintenance	Paclitaxel and carboplatin	Paclitaxel, carboplatin and paclitaxel management	50% (2‐y PFS)	54% (2‐y PFS)	99	1.3	1.1
12	Hoskins 2010^39^	First‐line	Paclitaxel and carboplatin	Sequential cisplatin and topotecan and paclitaxel and carboplatin	16 mo (median)	16.2 mo	410	1.25	1.0
13	Lhomme 2008^40^	First‐line	Paclitaxel and carboplatin	Paclitaxel, carboplatin, and valspodar	18 mo (median)^a^	13.5 mo^a^	377	1.3	0.8
14	Hirte 2006^41^	First‐line maintenance	Observation	Tanomastat	20 mo (median)	9.2 mo	121	1.4	0.5
15	De Placido 2004^42^	First‐line maintenance	Observation	Topotecan	18 mo (median)	28 mo	93	1.5	1.6
16	Ferrandina 2008^43^	Recurrence	Pegylated liposomal doxorubicin	Gemcitabine	2.8 mo (median)^a^	3.7 mo^a^	76	1.6	1.3
17	Berek 2004^44^	First‐line maintenance	Observation	Oregovomab	18 mo (median)^c^	10.8 mo^c^	72	1.5	0.6
18	Mobus 2007^17^	First‐line	Paclitaxel and carboplatin ± etoposide	High‐dose chemotherapy^d^	0.35 (2‐y PFS) 16.8 mo (median)	20.5 mo	89	1.4	1.1
19	Vergote 2010^45^	Recurrence	Pegylated liposomal dxorubicin	Canfosfamide and pegylated liposomal doxorubicin	3.5 mo	3.7 mo	60	1.5	1.1
20	Reed 2006^46^	First‐line	Treosulfan	Carboplatin	9.2 mo	5.0 mo	102	1.5	0.5

Abbreviations: A/E, actual‐over‐expected ratio; PFS, progression‐free survival; TTP, time to disease progression.

Detailed is expected PFS/TTP of the control arm used for statistical calculations, actual PFS/TTP achieved by this arm as well as size effect expected from the experimental treatment. Finally A/E ratio for PFS/TTP of the control arm is given. A/E ratios were rounded to one decimal place.

a Used TTP as progression measure.

b Third chemotherapy agent included gemcitabine, pegylated liposomal doxorubicin, or topotecan.

c Used time to disease recurrence as a progression measure.

d High‐dose chemotherapy includes 2 cycles of fortnightly paclitaxel and cyclophosphamide with peripheral blood stem cell harvest followed by 3 cycles of carboplatin and paclitaxel with melphalan included in the final cycle. Both arms have the option of etoposide and up to 4 cycles of maintenance topotecan.

### Comparison of the Accuracy of Predictions of Survival‐Based And Progression‐Based Endpoints

When compared with the 20 trials with a progression‐based primary outcome, the 15 trials with OS as a primary endpoint were found to be significantly more likely to underestimate the control arm outcome (Wilcoxon 2‐sample test, *P*<.001).

To account for potential random error introduced by the inclusion of trials with small sample sizes, the 2 groups were compared again after excluding those in which the control group sample size was <100 patients. A total of 3 and 7 studies were excluded from the OS^15^, ^23^, ^29^ and PFS^17^, ^36^, ^38^, ^42^, ^43^, ^44^, ^45^ analyses, respectively. This confirmed a statistically significant difference (*P* = .001). A secondary analysis was performed to account for the 2 trials in which the expected survival was updated at an interim analysis.^21^, ^24^ Even with the revised expected survival, there was statistically more underestimation noted among survival‐based trials than progression‐based trials (*P* = .002).

## DISCUSSION

The results of the current study demonstrate that for EOC trials published over the past decade there has been significant imprecision when estimating the control arm outcome in RP3 trials. This imprecision is present in trials with both progression‐based and survival‐based primary endpoints. However the data presented herein indicate that trials with an OS endpoint were significantly more likely to underestimate the outcome of the control arm than those using PFS or TTP as the endpoint.

A limitation of the current review is that relatively few RP3 trials met all eligibility criteria. Approximately one‐half of the EOC RP3 trials were excluded, in most instances because of missing information regarding sample size calculations. This finding is perhaps not surprising because it has previously been reported that information regarding sample size calculation is frequently missing in clinical trial publications.^47^ A further limitation is that some of the trials included had a small sample size, sometimes as a result of poor accrual, and as such are subject to random error. To minimize the potential impact of this confounding factor, a sensitivity analysis was performed excluding those trials with a small sample size. This demonstrated similar results to those observed in the analysis of all trials.

It is important to consider why OS was significantly underestimated when designing systemic therapy trials in EOC. It is possible that changing patterns of care and improvements in survival for patients with EOC during the years these trials were designed and conducted may have rendered historical data obsolete, causing investigators to underestimate the outcome anticipated with standard therapy. Improved surgical techniques and consequent stage migration may also have had an impact on OS estimations.

The time between the initial trial design and the completion of accrual typically spans many years. After the completion of accrual, more time elapses until enough events have occurred to analyze survival. Thus, even when factoring in differences in patterns of care between reported historical data and available care at the moment of study design, it is possible that evolving therapy during the conduct of the trial may further confound these estimations.

It should be noted that since 1990, paclitaxel, gemcitabine, and pegylated liposomal doxorubicin have demonstrated efficacy in patients with EOC and have been approved for treatment.^32^, ^39^, ^48^ Most recently, antiangiogenic agents have demonstrated effectiveness in EOC clinical trials.^7^, ^8^ The progressive introduction of these agents in trials and in routine care during the conduct of the majority of the EOC trials published within the last decade may well have caused actual survival to deviate from historical controls.

Moreover, for many of the clinical trials included in the current analysis, the experimental agents studied (eg, topotecan, anthracyclines, and gemcitabine) were commercially available either during or after the study was conducted, thereby raising the potential for off‐trial crossover with the experimental agent in a percentage of patients, further confounding, and possibly increasing observed OS in control arms.

As a consequence of the small sample size, our ability to delineate whether underestimation of OS is becoming more problematic with time is hindered, but one would expect this to be the case, reflecting increasing therapeutic options after disease progression and a longer time to accrue patients to trials. This issue is likely to be clinically relevant in both the first‐line and recurrent setting and should be considered in the design of future research.

It is perhaps not surprising that such a prominent underestimation of outcome is not observed when trials use a progression‐based endpoint. In contrast to PFS, OS is a composite of both PFS and survival after disease progression.^49^ Time from treatment initiation until either disease recurrence or progression is not influenced by post‐trial treatment nor crossover, making historical publications regarding the efficacy of a single treatment more reliable than estimates of survival that reflect a sequence of treatments that dynamically evolve as new drugs become available.

When patients with metastatic disease such as ovarian cancer develop disease progression, there are several potential interventions available including: 1) crossover; 2) treatment with an alternative agent; 3) continuation with the same agent if there is symptomatic benefit; or 4) no further therapy.^49^ The heterogeneity of these options makes it difficult to assess the influence (if any) of the initial randomized therapy on OS due to the confounding and diluting effect from each subsequent intervention.^49^ Fewer variables come into play when estimating TTP or PFS than when estimating time to death. Moreover, the time to the event is shorter, thereby leading to more predictable estimates.

The finding that OS has been underestimated when designing EOC trials has potentially important implications both for interpreting recently published trials and for designing future trials. A significant underestimation of the anticipated control arm outcome means that the observed event rate will be lower than anticipated during the time the trial is being conducted. Because sample size is proportional to the square power of the difference in the event rate between the control and experimental arms, the trials become underpowered to detect a difference of the magnitude they were designed for. As the event rate decreases, the sample size will need to increase exponentially to demonstrate the same absolute difference in survival. Table 3 illustrates how relatively small changes in observed outcome and relative size effect studied affect the sample size required to maintain adequate statistical power to demonstrate the same benefit.

**Table 3:** Theoretical Scenarios to Illustrate How Variations in Survival of the Control Cohort Impact on the Sample Size Required to Maintain Statistical Power

Scenario	A/E Ratio	Expected Outcome of Control Arm	Size Effect Studied	Absolute Benefit in Survival to Demonstrate the Same Benefit	Expected Outcome of Experimental Arm	Sample Size Required
Initial trial design	Not applicable	24 mo	1.25	6 mo	30 mo	455 patients per arm
No. of patients to demonstrate same maintenance of absolute benefit	1.5	36 mo	1.166	6 mo	40 mo	1685 patients per arm
No. of patients to demonstrate maintenance of effect size	1.5	36 mo	1.25	9 mo	45 mo	495 patients per arm

Abbreviation: A/E ratio, actual‐over‐expected ratio.

Calculations were made assuming an accrual rate of 20 patients per month and a follow‐up time of 24 months for the first scenario and 36 months for the latter 2 scenarios.

Discrepancies between the expected and actual outcome of the control arm can potentially result in clinically relevant survival differences being missed because of a lack of sufficient statistical power. When the result of an endpoint is underestimated owing to lower event rates, the trial duration is likely to be longer and more expensive than anticipated. Consequently, it is concerning that EOC trials published within the past decade were potentially underpowered to demonstrate the magnitude of absolute survival benefit they were designed for despite being longer and potentially more costly.

The difficulty in predicting and detecting OS improvements has significant ramifications for regulatory agencies that rely on these trials and the accurate determination of the size of the benefit to make funding decisions. Furthermore, the underestimation of OS in most EOC trials published to date highlights the challenges in adequately designing trials that are powered to demonstrate differences in survival due to the number of variables requiring consideration. These challenges may be more problematic in malignancies with a longer survival and many available treatment options, such as EOC, low‐grade lymphomas, or breast cancer, compared with those with limited treatment options and short survival times such as metastatic pancreatic or lung cancer. In contrast, among diseases in which the median survival after disease progression has been classically shorter (ie, <12 months), such as advanced colorectal cancer and non‐small lung cancer, a stronger correlation between PFS and OS has been demonstrated.^49^, ^50^, ^51^ This may mean that OS is easier to predict in these diseases. Data regarding the accuracy of trial survival predictions in other disease sites need to be collected to formerly test this hypothesis. However, this report highlights that when designing a trial that is properly powered to address differences in OS in cancers with many treatment options, correctly estimating the control arm outcome can be challenging.

The data from the current study highlight the difficulty inherent in estimating the actual outcome of a cohort of patients. In EOC, this has led to an almost routine underestimation of expected survival in recently reported RP3 trials. These challenges should be addressed when designing future phase 3 trials in EOC as well as other malignancies. Severely underestimating the control arm outcome can lead to a trial being more complex and expensive to conduct than initially planned but remaining statistically underpowered to demonstrate the clinically meaningful survival difference it was designed to detect.

## FUNDING SUPPORT

No specific funding was disclosed.

## CONFLICT OF INTEREST DISCLOSURES

The authors made no disclosures.
